# Interaction of HPV16 and Cutaneous HPV in Head and Neck Cancer

**DOI:** 10.3390/cancers14215197

**Published:** 2022-10-23

**Authors:** Walid A. Al-Soneidar, Sam Harper, Babatunde Y. Alli, Belinda Nicolau

**Affiliations:** 1 Department of Epidemiology, Biostatistics and Occupational Health, McGill University, Montreal, QC H3A 1G1, Canada; 2 Faculty of Dentistry, McGill University, Montreal, QC H3A 1G1, Canada

**Keywords:** HPV, head and neck cancer, human papillomavirus, case–control studies

## Abstract

**Simple Summary:**

Infection with human papillomaviruses (HPV) increases the risk of developing head and neck cancer (HNC). There are over 200 known genotypes of HPV, but only a minority are carcinogenic. Cutaneous genotypes, which are traditionally isolated from the skin, were thought to be benign. However, recent evidence shows that they could be related to some cancers including HNC. Our goal in this study has been to investigate if there is an interaction between HPV16, a high-risk genotype from the alpha genus, with cutaneous HPV (beta and gamma genera) in HNC. We found preliminary evidence that among those infected with HPV16, co-infection with beta HPV could weaken the carcinogenic effect whereas coinfection with gamma HPV could strengthen the carcinogenic effect. While our findings show there might be an interaction between HPV16 and cutaneous HPV, these results are not conclusive and warrant further investigation. Future studies with larger sample sizes are needed.

**Abstract:**

Objectives: Human papillomavirus 16 (HPV16) is an established risk factor for Head and Neck Cancer (HNC). Recent reports have shown that genotypes from the beta (β) and gamma (γ) genera, also known as cutaneous HPV, can be found in the oral cavity, but their role is largely unidentified. We investigated the interaction between oral HPV16 and cutaneous HPV in HNC. Methods: We use data on incident HNC cases (*n* = 384) and frequency-matched hospital-based controls (*n* = 423) from the HeNCe Life study in Montreal, Canada. Participants were tested for alpha HPV and cutaneous genera using oral mouth rinse and brush samples. We used unconditional logistic regression to obtain adjusted odds ratios (aOR) and 95% confidence interval (CI) as a measure of the effect between HPV and HNC and assessed the interaction between HPV genotypes on the multiplicative and additive scales. Results: Prevalence of HPV infection was higher among cases (73%) than controls (63.4%), with cases more likely to be coinfected with more than a single genotype, 52.9% vs. 43.5%, respectively. Infection with HPV16 alone had a strong effect on HNC risk aOR = 18.2 [6.2, 53.2], while infection with any cutaneous HPV, but not HPV16, appeared to have the opposite effect aOR = 0.8 [0.6, 1.1]. The observed effect of joint exposure to HPV16 and any cutaneous HPV (aOR = 20.4 [8.3, 50.1]) was stronger than the expected effect based on an assumption of independent exposures but was measured with considerable imprecision. While the point estimate suggests a positive interaction between HPV16 and cutaneous HPV, results were imprecise with relative excess risk due to interaction (RERI) = 2.4 [−23.3, 28.2]. Conclusion: There could be biologic interaction between HPV16 and genotypes from cutaneous genera, which warrants further investigation. Although cutaneous HPVs are not usually found in tumor tissues, they are cofactors that could interact with HPV16 in the oral cavity and thus strengthen the latter’s carcinogenic effect.

## 1. Introduction

Human papillomavirus 16 (HPV16) as well as other genotypes of HPV known as high-risk genotypes are sexually transmitted viruses [1] and established causes of head and neck cancer (HNC), mainly in the oropharynx [2]. A recent report in the HPV-related literature by Agalliu et al. suggested that genotypes from β- and γ-HPV genera, also known as cutaneous HPV (as a result of being first discovered in skin warts), could also increase the risk of HNC [3]. While the link between cutaneous HPV and skin cancer is clear [4,5,6], there is limited evidence supporting an independent carcinogenic effect in the head and neck. In other words, it is not clear if these viruses are carcinogenic per se or have rather a more limited role in assisting or enhancing the carcinogenic effect of known high-risk genotypes from the α-genus.

Agalliu et al. showed an increase in the risk of HNC among those infected with cutaneous HPV even after adjusting for HPV16 status, suggesting an independent carcinogenic effect [3]. We, however, found weak evidence supporting an independent role for cutaneous HPV. In another work that has been recently published [7], we found that γ-HPV could slightly increase the risk of HNC, but the magnitude of the effect was small and was measured with imprecision for rare detection rate in oral samples. β-HPV, on the other hand, did not show a harmful effect and was more common among controls than cases [7]. Further, among genera isolated from tumor lesions, we found only genotypes from the high-risk α-genus but no genotypes from the β and γ genera.

A possible hypothesis, often referred to as “hit and run”, which originated from animal studies of papillomaviruses and skin cancer, had been suggested for the role of cutaneous HPV [8,9]. According to this hypothesis, cutaneous HPV could play a role in the early stages of skin cancer by strengthening the role of high-risk genotypes and then disappear once carcinogenesis has been established. This explanation would be consistent with the observation that copies of β-HPV are more available in premalignant HPV than in established squamous cell carcinoma of the skin [10].

In this paper, we aimed to investigate the role and possible interaction between genotypes from different HPV genera in HNC development. We measured coinfection and the potential combined effect of HPV16, the most implicated HPV genotype in HNC, and β- and γ-HPV genera. More generally, we also investigated the interaction between high-risk α-HPV genotypes, as defined by the international association for research on cancer (IARC) [2], and infection with the β- and γ-genera.

## 2. Methods

### 2.1. Study Population

We used a hospital-based case–control design from the Canada site of the multi-country Head and Neck Cancer Life Study (HeNCe). Details on eligibility and study population can be found in previous publications [11,12,13]. Briefly, incident HNC cases and hospital-based controls frequency-matched by sex and age (within five years) were recruited from four major referral hospitals in Montreal, Canada. Data collection took place from September 2005 to November 2013. The study was designed to represent the catchment area of the Montreal metro area in Quebec—i.e., people who would be treated in either of the four hospitals should they develop HNC. Controls were selected from patients with non-chronic diseases attending outpatient clinics from which the cases were recruited. Ethical approval for HeNCe was obtained from McGill University and all participating hospitals.

To be included in the study, participants needed to: (a) be born in Canada; (b) speak either English or French (c) be at least 18 years old; (d) have no history of cancer, immunosuppressive condition, or mental disorder; and (e) live within 50 km of the hospital. This precaution was taken to ensure that treatment could not interfere with the patients’ biomedical status. A total of 460 HNC cases and 458 controls were recruited into the HeNCe Canada study. Among these participants, 818 individuals (389 cases and 429 controls) were tested for the presence of α-HPV. Because of resource limitations that prevented β-HPV and γ-HPV testing of all samples, we prioritized β-HPV testing. β-HPV testing was performed on oral cell samples of 824 participants (391 cases and 433 controls), while 544 were tested for γ-HPV (246 cases and 298 controls). To test for the interaction between HPV16 or α-HPV with β-HPV, we used the data for those tested for both α- and β-HPV (384 cases and 424 controls). Likewise, to test for interaction between HPV16 or α-HPV with γ-HPV, we used data from participants tested for both genera (242 cases and 294 controls). We also investigated interaction in the sample tested for all HPV genera (241 cases and 294 controls).

### 2.2. Data Collection

We used face-to-face interviews and a life-grid questionnaire to collect information from patients, which reduces the possibility of recall bias [14,15]. The questionnaire covered several domains such as sociodemographic characteristics, lifetime history of tobacco and alcohol use, and sexual behavior. Information on the cancer site, stage, and clinical condition was obtained from medical charts. The medical information on controls was used to confirm the stated reason for the visit in the interview. Using an OralCDx^®^ brush and mouthwash, epithelial cells were collected from various sites in the mouth, including the tumor site for cases, for HPV testing.

### 2.3. Oral HPV Testing

The HPV testing methodology for the HeNCe Life Study has been previously described [7,16]. Cell suspensions were centrifuged at 13,000× *g* for 15 min at 22 °C. Pellets were resuspended in 300 μL of 20 mmol/L Tris buffer (pH 8.3) and DNA was purified using the MasterPure™ Kit (Epicenter). Extracted DNA was kept frozen until tested with PCR at −70 °C. We used several molecular techniques for testing and genotyping HPV genera. All HPV DNA samples were tested for β-globin, with positive samples considered for further HPV genotyping and negative samples considered inadequate for PCR. A polymerase chain reaction (PCR) assay with PGMY09-PGMY11-primers and linear array was used to test for α-HPV genotypes. This is capable of detecting 36 mucosal genotypes. To detect cutaneous genotypes, β- and γ-HPV, we used multiplex PCR with bead-based Luminex technology. This technique is capable of detecting 43 β-HPV genotypes and 52 γ-HPV genotypes [17].

### 2.4. Outcome Variable

The outcome of interest is incident Head and Neck Cancer (HNC). All cases were incident cancer cases, which represent the first cancer a patient develops and before they receive any treatment. This precaution was taken to ensure that chemotherapy or any other treatment could not interfere with the patients’ biomedical status. Cases ranged from carcinoma in situ to stage IV cancer and were all defined according to the International Classification of Diseases version 10 as follows: oral cavity cancers (OCC) (C02-C06), oropharyngeal cancer (OPC) (C01, C02.4, C05.01, C05.2, C09, C10, C13) and laryngeal cancers (LC) (C32). Cancers of the lip, nasopharyngeal cancers, and salivary glands were excluded due to their different etiologies.

### 2.5. Exposure Variables

We investigated interactions between the following combination of exposures: HPV16 vs. any γ-HPV types; HPV16 vs. any β-HPV types; HPV16 vs. any β- or γ-HPV types; and any high-risk HPV vs. any β- or γ-HPV types. In all models, we controlled for confounding for the main exposure with the outcome and used the same sufficient set of confounders for both exposures which include age, sex, tobacco smoking, and alcohol use.

### 2.6. Statistical Analysis

We used unconditional logistic regression analysis adjusting for confounding (age, sex, smoking, and alcohol consumption), and assumed an identical set of confounders for all genotypes, meaning that the minimum sufficient set needed to block the back door path for the first exposure is identical to the minimum sufficient set needed for the second exposure [18]. We evaluated interaction on the multiplicative scale by including a cross-product (interaction term) between the two exposures of interest, which when exponentiated represents the ratio of the odds ratios (OR_11_/OR_10_ OR_01_). We also measured interaction on the additive scale (biologic interaction) using three indices—Relative Excess Risk due to Interaction (RERI), Attribution Proportion (AP), and Synergy Index (S) as suggested by Rothman [18,19]. Each of these measures has advantages and limitations beyond the scope of this paper, and we present them all here as recommended in the literature [18,20,21].

RERI represents the difference between the observed joint effect of multiple exposures and the expected joint effect based on the individual independent effects of the two exposures under consideration (calculated as OR_11_ − OR_01_ − OR_10_ + 1). While a RERI of zero indicates perfect additivity (i.e., no interaction), a value of greater or less than zero indicates positive or negative additive interaction, respectively [22]. In addition, we estimated the AP as RERI/OR_11_, which ranges from −1 to +1. AP equals zero if there is no interaction, is larger than zero in the case of positive interaction, and less than zero in the case of negative interaction. The SI is the ratio of the combined effects and the individual effects and ranges from zero to infinity. If SI equals one, it indicates an absence of interaction. However, larger or smaller numbers indicate a departure from additivity (>1.0 = positive interaction; <1.0 = negative interaction). Wald-type 95% confidence intervals (CI) were estimated for all individual and joint estimates with 95% CI estimated for the measures of additive interaction using the delta method [23]. All interaction analyses, measures of interaction, and effect estimates are reported in two-by-two tables as recommended by Knol and VanderWeele [20]. Tables were created with *InteractionR* package [24] in R version 4.1.1, while graphs showing adjusted predicted probability of HNC for each level of the interaction between α-HPV and each of β and γ HPV were created with Stata/MP version 16.1 (StataCorp, College Station, TX, USA). Analysis code can be found on Open Science Framework (https://osf.io/nsqut/?view_only=d7e34009d76c4b669816038960aded42 (accessed on 16 October 2022)).

## 3. Results

Table 1 describes the relevant characteristics of the participants tested for all genera and stratified by case–control status. By design, most of the cases and controls were men, with no difference in age in both groups—approximately 61 years of age. However, a relatively higher proportion of cases were tobacco smokers compared to controls, and the smokers among cases were heavier smokers throughout life compared to the smokers in the control group (47 pack-years vs 32 pack-years) (Table 1). A much higher proportion of the cases were HPV16 positive compared to controls (27% vs. 2%). For cutaneous HPV, infection with any γ-HPV was more common in cases (34% vs. 25%), while any β-HPV had a similar prevalence between cases and controls (59% vs. 60%). Prevalence of HPV infection overall was higher among cases than controls, with cases more likely to be coinfected with more than a single genotype, respectively.

**Figure 1:**
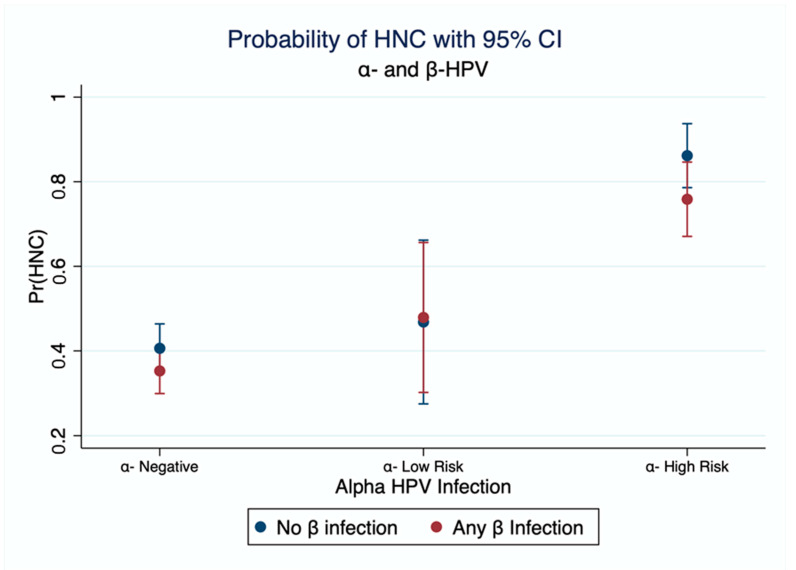
Interaction between α-HPV and β-HPV Marginal estimation of probability of HNC. from a logistic model with interaction between α-HPV and β-HPV. High Risk genotypes as identified by IARC: HPV16, 18, 31, 33, 35, 39, 45, 51, 52, 56, 58, and 59. Model is adjusted for age, sex, smoking, and alcohol.

As expected, infection with HPV16 in the absence of β-HPV had a strong effect on HNC OR = 20.9 [7.2, 60.6], while infection with any β-HPV but no HPV16 appeared protective OR = 0.7 [0.5, 1.0] (Table 2). The risk of HNC among those infected with both HPV16 and any β-HPV genotype is lower than it is among those exposed to HPV16 alone OR = 16.9 [6.9, 41.5]. All measures of additive interaction are consistent and show a negative interaction (RERI < 0, AP < 0; SI < 1). Figure 1 shows the marginal predicted probability of HNC as estimated from the logistic model with interaction between α-HPV and β-HPV. There is an increase in the probability of HNC when infected with low-risk α-HPV and an even higher probability of HNC when infected with high-risk α-HPV. Co-infection with β-HPV shows a slight reduction in risk when coinfection with high-risk genotypes (Figure 1), although with an overlap in confidence limits.

**Figure 2:**
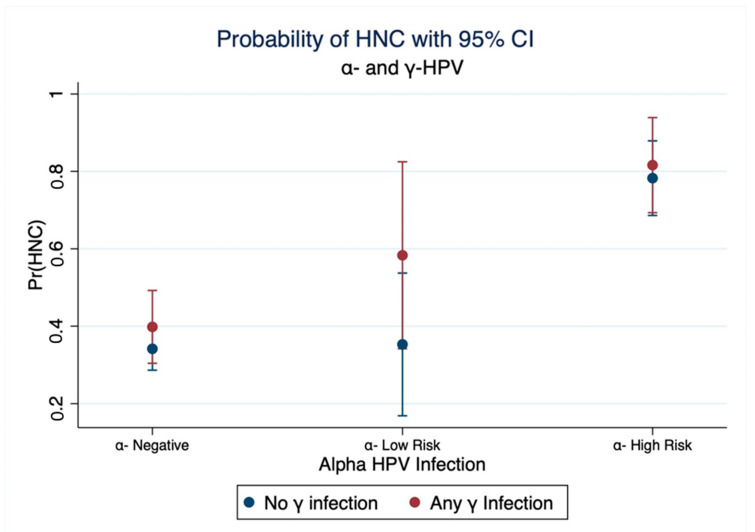
Interaction between α-HPV and γ-HPV. Marginal estimation of probability of HNC from a logistic model with interaction term between α-HPV and γ-HPV. Model is adjusted for age, sex, smoking, and alcohol.

Table 3 shows that infection of HPV16 in the absence of γ-HPV had a strong effect on HNC OR = 18.5 [6.8, 50.6]. There was a weak relation between infection with any genotype from the γ-genus without HPV16 and HNC OR = 1.1 [0.7, 1.7]. Those coinfected with both HPV16 and any γ-HPV had a stronger effect on HNC than being infected with one but not both genera OR = 36 [8.1, 160.8], but measured with considerable imprecision. The marginal predicted probability of HNC shows that coinfection with any γ-HPV may increase the risk of HNC among those infected with α-HPV (Figure 2). Additive interaction between HPV16 and γ-HPV indicates that the excess risk on the odds ratio scale that is attributed due to interaction (RERI) is 17.44 [−38.34, 73.21] and that 48% of the effect among those jointly exposed to HPV16 and any γ-HPV is due to interaction (AP = 0.48 [−0.41, 1.38]). The direction of measures of additive interaction may suggest a positive mechanistic interaction between HPV16 and γ-HPV, although results are imprecise due to the small sample size.

AppendixTable A1 shows that infection with HPV16 in the absence of any cutaneous genotype (β and γ-HPV) had a strong effect on HNC risk adjusted OR = 18.2 [6.2, 53.2], while the effect of infection with any cutaneous HPV alone on HNC risk is OR = 0.8 [0.6, 1.1]. The joint effect of being infected with HPV16 and any cutaneous HPV was stronger than the effect of either one alone 20.4 [8.3, 50.1]. As expected, when the analysis was restricted to oropharyngeal cancer (OPC) (Appendix Table A2), the effect of HPV16 alone on the risk of the OPC was more pronounced OR = 40.1 [13.3, 121.0], and while being infected with any cutaneous HPV alone on the risk of OPC appears to be protective OR = 0.7 [0.5, 1.1], coinfection with HPV16 and any cutaneous HPV leads to a higher risk for OPC than being infected with either exposure alone OR = 54 [19.6, 148.5]. All measures of additive interaction suggest a positive interaction between HPV16 and cutaneous HPV.

## 4. Discussion

Interaction happens when one variable’s effect on an outcome varies or interacts depending on the value of another variable [25]. Using a sufficient causal framework, interaction takes place when two exposures are both components of the same sufficient cause, meaning that for some individuals in the population the two exposures need to be present for the outcome to develop [18,26], and will not experience the outcome in the absence of both exposures. In this study, we evaluated, the role of cutaneous HPV as a potentially important cofactor for the development of HNC of viral etiology. To the best of our knowledge, this is the first epidemiolocal evidence studying the interaction of HPV genera in the HNC literature. While our results were imprecise, we report a preliminary view of the possible interaction between cutaneous HPV and HPV16, and this interaction is more profound in the oropharynx. The interaction seems to go in different directions depending on the genus of cutaneous HPV as co-infection with β-HPV created a negative interaction whereas co-infection with γ-HPV created a positive interaction.

Having positive interaction on the additive scale means that for some individuals in the sample, the presence of two of these risk factors, for example, α-HPV and HPV16, needs to happen for cancer to develop. However, in case–control studies, we can only infer the direction of interaction, but not the magnitude as baseline risks are unknown [21]. VanderWeele argues that if RERI > 1 we can assume that there is a sufficient cause interaction between the two exposures, when RERI > 2 as was the case for HPV16 and γ-HPV and HPV and any cutaneous HPV, exposures can have *epistatic* interaction [27]. This means there are individuals in the population who will have the outcome (HNC) if and only if both exposures are present. While the point estimate of RERI for the interaction between HPV16 and γ-HPV is indeed > 2, the wide confidence interval makes concluding epistatic interaction impossible.

The results above support the “hit and run” hypothesis stemming from animal studies of HPV genotypes and carcinogenesis [8,9]. According to this hypothesis, cutaneous HPV genotypes promote and assist the carcinogenic effect of mucosal HPV (α-HPV) but are not necessarily carcinogenic in themselves in the oral mucosa. Combining the evidence from this study and what has been reported in animal models, strengthens the hypothesis that the carcinogenicity of HPV16 and high-risk genotypes from the α-genus is increased in case of coinfection with cutaneous HPV. However, reaching a conclusion on the dynamics and interplay between these viruses is difficult from studies looking at HPV infection in a snapshot rather than over time.

A limitation of this study is the small sample size which leads to large imprecision in our interaction estimates. This is because cutaneous HPVs, especially γ-HPV, have a low prevalence in the population [28,29]. As a result, we could not estimate the interaction between HPV16 and specific genotypes from the β- or γ-HPV genera. Additionally, our interaction estimates on the additive and multiplicative scales have wide confidence intervals. Although our study is one of the largest case–control studies of HNC in Canada, HeNCe was not designed for interaction analysis, and we were limited by the logistic and cost of genotyping cutaneous HPV. Future studies should consider planning combining data from several studies, for example in a consortium, as large sample sizes are warranted to quantify interaction [30].

## 5. Conclusions

Results in this study indicate that while HPV16 remains a strong risk factor for HNC, the role of cutaneous genotypes is less conclusive. There could be an interaction between HPV genera with unknown biological mechanisms. Further biological and epidemiological studies with larger samples are warranted to elucidate the mechanism of interaction that could occur between HPV genera and could play a role in carcinogenesis. We conclude that while α-HPV is still the main player in HNC development, the role of cutaneous HPV as a helping factor should be considered.

## Figures and Tables

**Table 1:** Distribution of sociodemographic and behavior characteristics among cases and controls.

	Controls	Cases
	*n* = 294	*n* = 241
Age (mean (SD))	60.73 (11.06)	60.91 (10.34)
20–39 years	9 (3.1)	5 (2.1)
40–49 years	34 (11.6)	24 (10.0)
50–59 years	94 (32.0)	89 (36.9)
60–69 years	93 (31.6)	72 (29.9)
70–79 years	51 (17.3)	44 (18.3)
80 years and above	13 (4.4)	7 (2.9)
Sex (%)		
Female	93 (31.6)	60 (24.9)
Male	201 (68.4)	181 (75.1)
		
Years of education (mean (SD))	13.95 (4.24)	12.37 (3.85)
		
Ever smoker (%)		
Ever	214 (72.8)	207 (85.9)
Never	80 (27.2)	34 (14.1)
Smoking pack-years (mean (SD))	32.32 (44.79)	46.50 (41.64)
		
Ever drinker		
Ever	248 (84.4)	206 (85.5)
Never	46 (15.6)	35 (14.5)
Ethanol liter-years (mean (SD))	450.32 (972.15)	855.93 (1688.93)
		
HPV16 (%)		
Negative	287 (97.6)	177 (73.4)
Positive	7 (2.4)	64 (26.6)
		
Any beta HPV (%)		
Negative	118 (40.1)	98 (40.7)
Positive	176 (59.9)	143 (59.3)
		
Any gamma HPV (%)		
Negative	222 (75.5)	159 (66.0)
Positive	72 (24.5)	82 (34.0)

**Table 2:** Interaction between Infection with HPV16 and infection with any β-HPV genotype.

Interaction of HPV16 and Beta HPV					
	Beta HPV Absent			Beta HPV Present	Effect of Beta HPV within the Strata of HPV16
	N cases/controls	OR [95% CI]	N cases/controls	OR [95% CI]	OR [95% CI]
HPV16 absent	140/183	1 [Reference]	137/230	0.73 [0.53, 1.01]	0.73 [0.53, 1.01]
HPV16 present	51/4	20.86 [7.19, 60.56]	54/6	16.87 [6.85, 41.54]	0.81 [0.21, 3.1]
Effect of HPV16 within the strata of Beta HPV		20.86 [7.19, 60.56]		23.05 [9.35, 56.82]	
					
Multiplicative scale		1.1 [0.28, 4.4]			
RERI		−3.72 [−29.7, 22.26]			
AP		−0.22 [−1.87, 1.43]			
SI		0.81 [0.19, 3.38]			

ORs are adjusted for age, sex, smoking, and alcohol consumption. Values in parentheses are 95% confidence intervals. Abbreviations: RERI, relative excess risk due to interaction; AP, attributable proportion; SI, synergy index. Multiplicative interaction (OR11/OR01 * OR10) is measured from a logistic model as and exponent of the coefficient of the interaction term of the two variables e^(β(A * B))^.

**Table 3:** Interaction between Infection with HPV16 and infection with any γ-HPV genotype.

Interaction of HPV16 and Gamma HPV					
	Gamma HPV absent		Gamma HPV present		Effect of Gamma HPV within the strata of HPV16
	N cases/controls	OR [95% CI]	N cases/controls	OR [95% CI]	OR [95% CI]
HPV16 absent	122/217	1 [Reference]	55/70	1.05 [0.67, 1.66]	1.05 [0.67, 1.66]
HPV16 present	37/5	18.5 [6.77, 50.57]	27/2	35.98 [8.05, 160.82]	1.95 [0.34, 11.15]
Effect of HPV16 within the strata of Gamma HPV		18.5 [6.77, 50.57]		34.25 [7.38, 158.85]	
					
Multiplicative scale		1.85 [0.3, 11.24]			
RERI		17.44 [−38.34, 73.21]			
AP		0.48 [−0.41, 1.38]			
SI		1.99 [0.33, 12.17]			

ORs are adjusted for age, sex, smoking, and alcohol consumption. Values in parentheses are 95% confidence intervals. Abbreviations: RERI, relative excess risk due to interaction; AP, attributable proportion; SI, synergy index. Multiplicative interaction (OR11/OR01 * OR10) is measured from a logistic model as and exponent of the coefficient of the interaction term of the two variables e^(β(A * B))^.

## Data Availability

The data underlying this article cannot be shared publicly due to the privacy of individuals that participated in the study and institutional restrictions.

## Appendix A

**Table A1:** Interaction between HPV16 and infection with any Cutaneous HPV (β or γ genotype).

Interaction of HPV16 and Any Beta or Gamma			
	Any Beta or Gamma Absent	Any Beta or Gamma Present	Effect of Any Beta or Gamma within the Strata of HPV16
	OR [95% CI]	OR [95% CI]	OR [95% CI]
HPV16 absent	1 [Reference]	0.82 [0.59, 1.14]	0.82 [0.59, 1.14]
HPV16 present	18.15 [6.19, 53.24]	20.42 [8.32, 50.11]	1.12 [0.29, 4.32]
Effect of HPV16 within the strata of Any Beta or Gamma	18.15 [6.19, 53.24]	24.78 [10.14, 60.58]	
Multiplicative scale	1.37 [0.34, 5.46]		
RERI	2.44 [−23.27, 28.15]		
AP	0.12 [−1.07, 1.31]		
SI	1.14 [0.27, 4.78]		

ORs are adjusted for age, sex, smoking, and alcohol consumption. Values in parentheses are 95% confidence intervals. Abbreviations: RERI, relative excess risk due to interaction; AP, attributable proportion; SI, synergy index. Multiplicative interaction (OR_11_/OR_01_ * OR_10_) is measured from a logistic model as and exponent of the coefficient of the interaction term of the two variables e^(β(A * B))^.

## Appendix B

**Table A2:** Interaction between HPV16 and infection with any Cutaneous HPV In Oropharynx.

Interaction of HPV16 and Any Cutaneous HPV (Oropharyngeal Cancers Only)			
	Any Cutaneous HPV Absent	Any Cutaneous HPV Present	Effect of Any Cutaneous HPV within the Strata of HPV16
	OR [95% CI]	OR [95% CI]	OR [95% CI]
HPV16 absent	1 [Reference]	0.71 [0.45, 1.13]	0.71 [0.45, 1.13]
HPV16 present	40.13 [13.31, 120.99]	53.9 [19.56, 148.51]	1.34 [0.33, 5.44]
Effect of HPV16 within the strata of Any Cutaneous HPV	40.13 [13.31, 120.99]	75.96 [27.68, 208.43]	
Multiplicative scale	1.89 [0.43, 8.27]		
RERI	14.07 [−51.65, 79.78]		
AP	0.26 [−0.78, 1.3]		
SI	1.36 [0.32, 5.73]		

ORs are adjusted for age, sex, smoking, and alcohol consumption. Values in parentheses are 95% confidence intervals. Abbreviations: RERI, relative excess risk due to interaction; AP, attributable proportion; SI, synergy index. Multiplicative interaction (OR_11_/OR_01_ * OR_10_) is measured from a logistic model as and exponent of the coefficient of the interaction term of the two variables e^(β(A * B))^.

## References

[1] Vaccarella S., Franceschi S., Herrero R., Muñoz N., Snijders P.J.F., Clifford G.M., Smith J.S., Lazcano-Ponce E., Sukvirach S., Shin H.-R. (2006). Sexual Behavior, Condom Use, and Human Papillomavirus: Pooled Analysis of the IARC Human Papillomavirus Prevalence Surveys. Cancer Epidemiol. Prev. Biomark..

[2] Westra W.H., Lewis J.S. (2017). Update from the 4th Edition of the World Health Organization Classification of Head and Neck Tumours: Oropharynx. Head Neck Pathol..

[3] Agalliu I., Gapstur S., Chen Z., Wang T., Anderson R.L., Teras L., Kreimer A.R., Hayes R.B., Freedman N.D., Burk R.D. (2016). Associations of Oral α-, β-, and γ-Human Papillomavirus Types With Risk of Incident Head and Neck Cancer. JAMA Oncol..

[4] Tampa M., Mitran C.I., Mitran M.I., Nicolae I., Dumitru A., Matei C., Manolescu L., Popa G.L., Caruntu C., Georgescu S.R. (2020). The Role of Beta HPV Types and HPV-Associated Inflammatory Processes in Cutaneous Squamous Cell Carcinoma. J. Immunol. Res..

[5] Tommasino M. (2019). HPV and Skin Carcinogenesis. Papillomavirus Res..

[6] Sichero L., Rollison D.E., Amorrortu R.P., Tommasino M. (2019). Beta Human Papillomavirus and Associated Diseases. Acta Cytol..

[7] Al-Soneidar W.A., Harper S., Madathil S.A., Schlecht N.F., Nicolau B. (2022). Do Cutaneous Human Papillomavirus Genotypes Affect Head and Neck Cancer? Evidence and Bias-Correction from a Case-Control Study. Cancer Epidemiol..

[8] Smith K.T., Saveria Campo M. (1988). “Hit and Run” Transformation of Mouse C127 Cells by Bovine Papillomavirus Type 4: The Viral DNA Is Required for the Initiation but Not for Maintenance of the Transformed Phenotype. Virology.

[9] Viarisio D., Müller-Decker K., Accardi R., Robitaille A., Dürst M., Beer K., Jansen L., Flechtenmacher C., Bozza M., Harbottle R. (2018). Beta HPV38 Oncoproteins Act with a Hit-and-Run Mechanism in Ultraviolet Radiation-Induced Skin Carcinogenesis in Mice. PLOS Pathog..

[10] Weissenborn S.J., Nindl I., Purdie K., Harwood C., Proby C., Breuer J., Majewski S., Pfister H., Wieland U. (2005). Human Papillomavirus-DNA Loads in Actinic Keratoses Exceed Those in Non-Melanoma Skin Cancers. J. Investig. Dermatol..

[11] Laprise C., Madathil S.A., Schlecht N.F., Castonguay G., Soulières D., Nguyen-Tan P.F., Allison P., Coutlée F., Hier M., Rousseau M.-C. (2017). Human Papillomavirus Genotypes and Risk of Head and Neck Cancers: Results from the HeNCe Life Case-Control Study. Oral Oncol..

[12] Farsi N.J., Rousseau M.-C., Schlecht N., Castonguay G., Allison P., Nguyen-Tan P.F., Souliéres D., Coutlée F., Hier M., Madathil S. (2017). Aetiological Heterogeneity of Head and Neck Squamous Cell Carcinomas: The Role of Human Papillomavirus Infections, Smoking and Alcohol. Carcinogenesis.

[13] Durán D., Al-Soneidar W.A., Madathil S.A., Kaufman J.S., Nicolau B. (2020). Quantitative Bias Analysis of Misclassification in Case-Control Studies: An Example with Human Papillomavirus and Oropharyngeal Cancer. Community Dent. Health.

[14] Berney L.R., Blane D.B. (1997). Collecting Retrospective Data: Accuracy of Recall after 50 Years Judged against Historical Records. Soc. Sci. Med..

[15] Holland P., Berney L., Blane D., Davey-Smith G. (1999). The Lifegrid Method in Health Inequalities Research. Health Var..

[16] Al-Soneidar W.A., Harper S., Coutlée F., Gheit T., Tommasino M., Nicolau B. (2022). Distribution of Alpha, Beta, and Gamma Human Papillomaviruses (HPV) in Oral Cell Samples and Relation to Sexual Behavior and Oral Health.

[17] Gheit T., Billoud G., de Koning M.N.C., Gemignani F., Forslund O., Sylla B.S., Vaccarella S., Franceschi S., Landi S., Quint W.G.V. (2007). Development of a Sensitive and Specific Multiplex PCR Method Combined with DNA Microarray Primer Extension To Detect Betapapillomavirus Types. J. Clin. Microbiol..

[18] Lash T.L., VanderWeele T.J., Haneuse S., Rothman K.J. (2021). Modern Epidemiology.

[19] Rothman K.J. (2012). Epidemiology: An Introduction.

[20] Knol M.J., VanderWeele T.J. (2012). Recommendations for Presenting Analyses of Effect Modification and Interaction. Int. J. Epidemiol..

[21] VanderWeele T.J. (2015). Explanation in Causal Inference: Methods for Mediation and Interaction.

[22] VanderWeele T.J., Knol M.J. (2014). A Tutorial on Interaction. Epidemiol. Methods.

[23] Hosmer D.W., Lemeshow S. (1992). Confidence Interval Estimation of Interaction. Epidemiology.

[24] Alli B.Y. (2021). InteractionR: An R Package for Full Reporting of Effect Modification and Interaction. Softw. Impacts.

[25] Szklo M., Nieto F.J. (2019). Epidemiology: Beyond the Basics.

[26] VanderWeele T.J., Robins J.M. (2007). The Identification of Synergism in the Sufficient-Component-Cause Framework. Epidemiology.

[27] VanderWeele T.J. (2010). Epistatic Interactions. Stat. Appl. Genet. Mol. Biol..

[28] Wong M.C.S., Vlantis A.C., Liang M., Wong P.Y., Ho W.C.S., Boon S.S., Sze R.K.H., Leung C., Chan P.K.S., Chen Z. (2018). Prevalence and Epidemiologic Profile of Oral Infection with Alpha, Beta, and Gamma Papillomaviruses in an Asian Chinese Population. J. Infect. Dis..

[29] Winer R.L., Gheit T., Feng Q., Stern J.E., Lin J., Cherne S., Tommasino M. (2019). Prevalence and Correlates of β– and γ–Human Papillomavirus Detection in Oral Samples From Mid-Adult Women. J. Infect. Dis..

[30] VanderWeele T.J. (2012). Sample Size and Power Calculations for Additive Interactions. Epidemiol. Methods.

